# The −KTS isoform of *Wt1* induces the transformation of Leydig cells into granulosa-like cells

**DOI:** 10.1038/s41421-024-00732-6

**Published:** 2024-11-12

**Authors:** Changhuo Cen, Bowen Liu, Limei Lin, Zhiming Shen, Nan Wang, Liangjun Zhang, Kai Meng, Min Chen, Fei Gao

**Affiliations:** 1 State Key Laboratory of Stem Cell and Reproductive Biology, Institute of Zoology, Chinese Academy of Sciences, Beijing, China; 2 Institute for Stem Cell and Regeneration, Chinese Academy of Sciences, Beijing, China; 3 University of Chinese Academy of Sciences, Beijing, China; 4 State Key Laboratory of Multimodal Artificial Intelligence Systems, Institute of Automation, Chinese Academy of Sciences, Beijing, China; 5 Lin He’s Academician Workstation of New Medicine and Clinical Translation, Jining Medical University, Jining, Shandong China; 6 Beijing Institute for Stem Cell and Regenerative Medicine, Beijing, China

**Keywords:** Reprogramming, Transdifferentiation

Dear Editor,

In mammals, the testis and ovary both originate from a common bipotential gonad primordium, the genital ridge, composed of multipotential somatic progenitor cells. Testicular development is initiated by testis-determining factors *Sry* and *Sox9*^1,2^, which induce the Sertoli cell lineage differentiation and formation of testicular cords. Conversely, in the absence of *Sry* in female gonads, the ovary-specific pathways including RSPO1/WNT4/β-catenin signaling^3^ and FOXL2^4^ commit the differentiation of pre-granulosa cells and direct ovarian fate. Leydig cells are steroidogenic cells crucial for spermatogenesis and maintaining secondary sex characteristics by producing steroid hormones in males.

The Wilms tumor gene (*Wt1*) is present in the entire urogenital ridges at embryonic day (E) 9.5, where it plays a regulatory role in the formation of the genital ridges and kidneys^5^. *Wt1* has two major isoforms, +KTS and −KTS, distinguished by the presence or absence of three amino acids (lysine-threonine-serine (KTS)) between the third and fourth zinc fingers. The +KTS variant has been identified as an essential regulator for *Sry* expression during sex determination. The –KTS isoform, while not necessary for sex determination, is crucial for the survival of the gonadal primordium^6,7^. A recent study published in *Science* reported that the *Wt1–KTS* isoform is also an ovary-determining factor, and its overexpression in male gonads, using a transgenic mouse model, leads to the transformation of Sertoli into granulosa cells^8^.

Our previous studies demonstrated that *Wt1* is required for lineage specification and maintenance of supporting cells in gonad development, and inactivation of *Wt1* results in Sertoli to Leydig-like cells transformation^9–11^. To investigate whether the overexpression of *Wt1* can induce the transformation of Leydig into Sertoli cells, a *Wt1–KTS* conditional transgenic mouse model (*Wt1*^*+/Ctg*^) was generated. The expression of the *Wt1–KTS* was controlled by the CAG promoter, with flox sites flanking a poly A stop codon inserted between the CAG promoter and coding sequence, and modulated by Cre activation (Supplementary Fig. S1a). The effectiveness of *Wt1–KTS* induction by Cre recombinase was evaluated using mouse embryonic fibroblast cells isolated from *Wt1*^*+/Ctg*^*;Cre-ER*^*TM*^ mice treated with tamoxifen. These results indicated a significant increase in both mRNA (Supplementary Fig. S1b) and protein (Supplementary Fig. S1c) levels of WT1–KTS after tamoxifen treatment, confirming the effective activation of *Wt1–KTS* by Cre recombinase.

To induce the overexpression of *Wt1–KTS* in undifferentiated gonadal somatic cells, *Wt1*^*+/Ctg*^ mice were bred with *Sf1-Cre* mice. It has been reported that *Sf1* (also known as *Nr5a1*) is expressed in the adrenogonadal primordium starting from E9.5^12^. Real-time PCR analysis demonstrated a significant increase in the mRNA level of *Wt1–KTS* in *Wt1*^*+/Ctg*^*;Sf1-Cre* testes at E12.5 (Supplementary Fig. S2a). Western blot results showed a substantial increase in the protein level of WT1 (Supplementary Fig. S2b, c). These findings confirmed the effective activation of *Wt1–KTS* in undifferentiated gonadal somatic cells mediated by *Sf1-Cre* during sex determination.

Adult *Wt1*^*+/Ctg*^*;Sf1-Cre* males were infertile, and the size of testes was significantly reduced (Fig. 1a). Histological analysis revealed well-organized seminiferous tubules in *Wt1*^*+/Ctg*^*;Sf1-Cre* mice, with SOX9-positive Sertoli cells located at the periphery region of seminiferous tubules, similar to control testes (Fig. 1a, black arrowheads). Interestingly, no 3β-HSD-positive Leydig cells were observed in *Wt1*^*+/Ctg*^*;Sf1-Cre* testes compared to the control (Fig. 1a, black arrows).

**Fig. 1:**
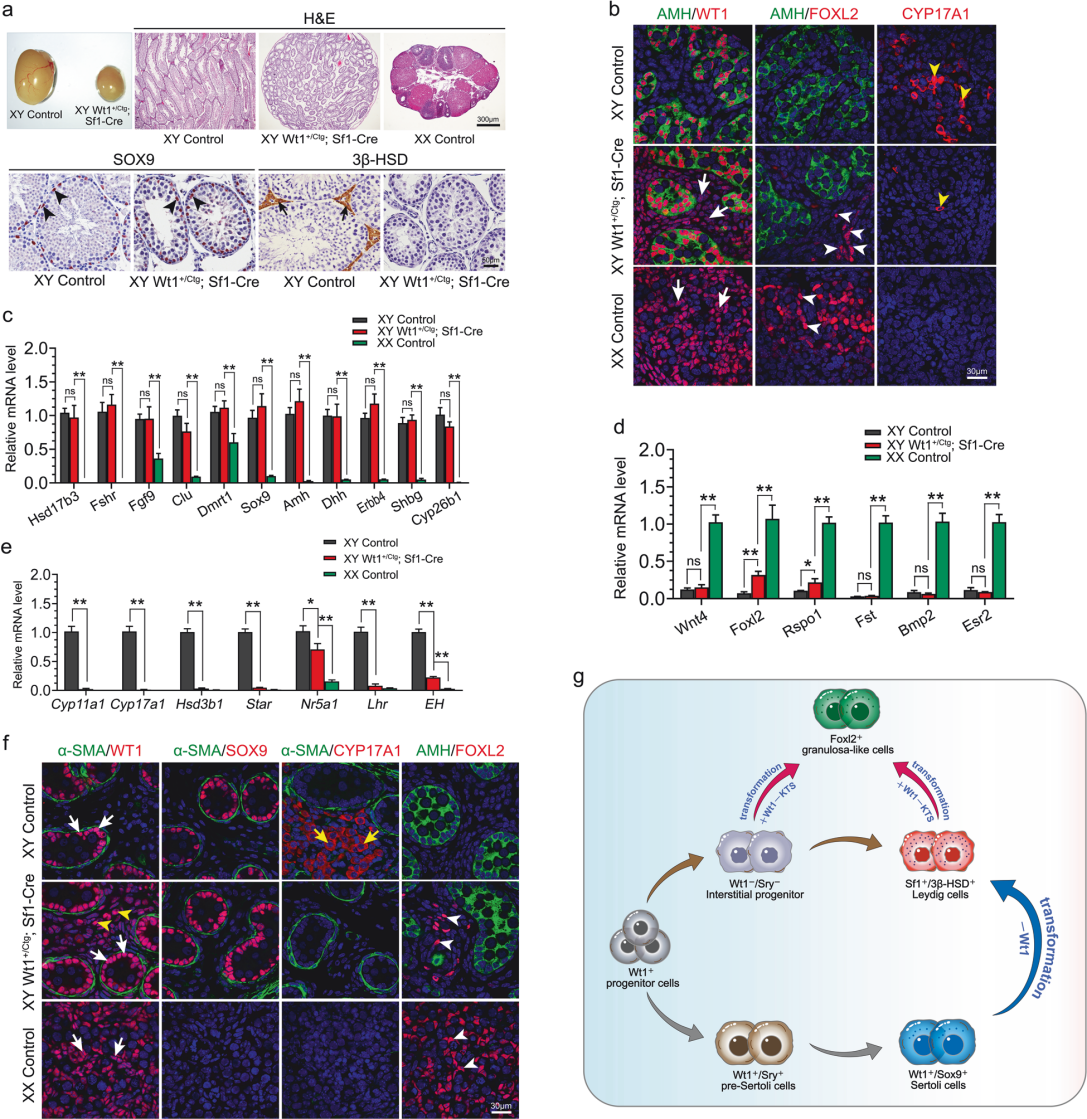
Overexpression of *Wt1–KTS* led to the transformation of Leydig to granulosa-like cells. **a** Histological analysis of gonads from control males, *Wt1*^*+/Ctg*^*;Sf1-Cre* males, and control females at 2 months of age. **b** Immunostaining of WT1, AMH, FOXL2, and CYP17A1 on control males, *Wt1*^*+/Ctg*^*;Sf1-Cre* males, and control females at E12.5. **c**–**e** The mRNA levels of Sertoli cell-specific genes (**c**), granulosa cell-specific genes (**d**), and Leydig cell-specific genes (**e**) in control testes, *Wt1*^*+/Ctg*^*;Sf1-Cre* testes and control ovaries at E12.5 (*n* = 6). **f** The expression of WT1, SOX9, CYP17A1, α-SMA, and FOXL2 in gonads of control males, *Wt1*^*+/Ctg*^*;Sf1-Cre* males, and control females at E16.5 was examined by immunofluorescence. **g** Schematic diagram illustrating the proposed role of *Wt1* in regulating gonadal somatic cell differentiation. Data are presented as mean ± SEM. Two-tailed Student’s *t*-test. *P < 0.05; **P < 0.01.

To further investigate the functions of *Wt1–KTS* in gonadal somatic cell differentiation during sex determination, the expression of Sertoli- and pre-granulosa cell-specific genes was examined at E12.5 by immunostaining and real-time PCR analyses. AMH, a specific marker for Sertoli cells, was expressed in the Sertoli cells of both control and *Wt1*^*+/Ctg*^*;Sf1-Cre* testes, with the structure of testicular cords being intact (Fig. 1b). FOXL2, abundantly expressed in control ovaries, was absent in control testes (Fig. 1b). However, FOXL2-positive cells were also observed in testes of *Wt1*^*+/Ctg*^*;Sf1-Cre* mice (Fig. 1b, white arrowheads), albeit in fewer number compared to a recent study^8^. Additionally, the WT1 signal in *Wt1*^*+/Ctg*^*;Sf1-Cre* testes was not only noted in AMH-positive Sertoli cells but also obviously detected in the interstitial cells (Fig. 1b, white arrowheads) situated in the testis interstitium, indicating the *Wt1–KTS* expression was significantly increased during sex determination. Furthermore, FOXL2-positive granulosa-like cells were located in the testis interstitium, distinct from AMH-positive Sertoli cells (Fig. 1b, white arrowheads). Meanwhile, the number of CYP17A1-positive (Fig. 1b, yellow arrowhead) Leydig cells was dramatically reduced in *Wt1*^*+/Ctg*^*;Sf1-Cre* testes. We further examined the expression of other Sertoli, granulosa, and Leydig cell-specific genes using real-time PCR in *Wt1*^*+/Ctg*^*;Sf1-Cre* testes. The mRNA levels of several Sertoli cell-specific genes, including *Hsd17b3*, *Fshr*, *Fgf9*, *Clu*, *Dmrt1*, *Sox9*, *Amh*, *Dhh*, *Erbb4*, *Shbg*, and *Cyp26b1*, remained unchanged in *Wt1*^*+/Ctg*^*;Sf1-Cre* testes compared to control (Fig. 1c). This suggests that the Sertoli cell differentiation was not affected by the overexpression of *Wt1–KTS* in gonadal somatic cells before sex determination. The mRNA levels of *Foxl2* and *Rspo1* were upregulated in *Wt1*^*+/Ctg*^*;Sf1-Cre* testes compared to control (Fig. 1d). However, the expression of other granulosa cell-specific genes, such as *Wnt4*, *Fst*, *Bmp2*, and *Esr2*, was not increased in the *Wt1*^*+/Ctg*^*;Sf1-Cre* testes (Fig. 1d). In line with the immunostaining results, the expression of steroidogenic cell-specific genes (e.g., *Cyp11a1*, *Cyp17a1*, *Hsd3b1*, *Star*, *Nr5a1*, *Lhr*, and *EH*) was obviously decreased (Fig. 1e).

To examine whether the Sertoli cells were transformed into granulosa-like cells at later developmental stages, the expression of *Sox9* and *Foxl2* was examined at E16.5 by immunostaining. Notably, seminiferous tubules outlined by peritubular myoid cell marker α-SMA and SOX9-positive Sertoli cells were observed at the periphery region of testicular cords in both control and *Wt1*^*+/Ctg*^*;Sf1-Cre* testes but not in control ovaries (Fig. 1f). Similar to the findings at E12.5, CYP17A1-positive Leydig cells were absent in *Wt1*^*+/Ctg*^*;Sf1-Cre* testes and control ovaries compared to control testes (Fig. 1f, yellow arrows). In addition, AMH was detected in the testicular cords of both control and *Wt1*^*+/Ctg*^*;Sf1-Cre* testes. FOXL2-positive granulosa cells (white arrowheads) were noted in the control ovary but absent in the control testis (Fig. 1f). By contrast, several FOXL2-positive granulosa-like cells (Fig. 1f, white arrowheads) were detected in the interstitium of *Wt1*^*+/Ctg*^*;Sf1-Cre* testes, distinct from the AMH signal. Importantly, FOXL2-positive granulosa-like cells were also observed in adult *Wt1*^*+/Ctg*^*;Sf1-Cre* testes, located outside of α-SMA-marked seminiferous tubules (Supplementary Fig. S3d, white arrows).

Our previous studies demonstrated that inactivation of *Wt1* causes supporting to steroidogenic cell transformation^9–11^. We hypothesized that the emergence of FOXL2-positive granulosa-like cells in *Wt1–KTS*-overexpressing testes might originate from Leydig cell progenitors. To test this hypothesis, we induced the overexpression of *Wt1–KTS*, specifically in Leydig cells, using *Cyp17a1-Cre* mice. To ascertain the specificity of *Cyp17a1-Cre*, we crossed these mice with *mT-mG* reporting mice. The results revealed that the GFP signal was exclusively detected in 3β-HSD-positive Leydig cells in *mT-mG;Cyp17a1-Cre* testes at E16.5 (Supplementary Fig. S4c, yellow arrows), indicating the specific activation of *Cyp17a1-Cre* in Leydig cells. Subsequently, we examined the expression of WT1 in control (*mT-mG;Cyp17a1-Cre*) and *Wt1*^*+/Ctg*^*;mT-mG; Cyp17a1-Cre* testes through immunostaining. In control testes, WT1 was observed in Sertoli cells, while no GFP and WT1 double-positive cells were detected (Supplementary Fig. S4f, white arrows). Conversely, in *Wt1*^*+/Ctg*^*;mT-mG;Cyp17a1-Cre* testes, WT1 was expressed not only in Sertoli cells but also in GFP-positive Leydig cells (Supplementary Fig. S4i, white arrowheads), suggesting the exclusive activation of *Cyp17a1-Cre* in Leydig cells. To analyze the fate of Leydig cells after *Wt1–KTS* overexpression, we examined the expression of 3β-HSD, SOX9, and FOXL2 by immunofluorescence at E16.5. In both control and *Wt1*^*+/Ctg*^*;mT-mG;Cyp17a1-Cre* testes, SOX9-positive Sertoli cells (yellow arrows) were observed within the seminiferous tubules, while no SOX9-positive cells were detected in control ovaries (Supplementary Fig. S5). The strong 3β-HSD signal was observed in the control testis interstitium and colocalized with the GFP signal (Supplementary Fig. S5b, white arrows), indicative of Leydig cells. Interestingly, a weaker 3β-HSD signal was detected in the interstitium of *Wt1*^*+/Ctg*^*;mT-mG;Cyp17a1-Cre* testes and colocalized with GFP signal (Supplementary Fig. S5e, white arrowheads). Notably, a small number of FOXL2-positive cells were observed in the interstitium of *Wt1*^*+/Ctg*^*;mT-mG;Cyp17a1-Cre* testes and colocalized with GFP signal (Supplementary Fig. S5f, yellow arrowheads). Real-time PCR analysis showed that the expression of Sertoli cell-specific genes (e.g., *Sox9*, *Amh*, *Dhh*, and *Fgf9*) was not significantly altered in *Wt1*^*+/Ctg*^*;Cyp17a1-Cre* testes, while the expression of steroidogenic cell-specific genes (e.g., *Cyp11a1*, *Star*, *Hsd3b1*, and *Cyp17a1*) was significantly downregulated. The mRNA levels of *Foxl2* and *Rspo1* were upregulated in *Wt1*^*+/Ctg*^*;Cyp17a1-Cre* testes, but other granulosa cell-specific genes (e.g., *Fst* and *Wnt4*) were not increased (Supplementary Fig. S5j). Furthermore, examination of *Wt1*^*+/Ctg*^*;mT-mG;Cyp17a1-Cre* testes at the adult stage revealed the absence of 3β-HSD-positive Leydig cells and the presence of FOXL2 and GFP double-positive cells (yellow arrows) in the interstitium (Supplementary Fig. S6), further confirming the transformation of Leydig into granulosa-like cells induced by *Wt1–KTS* overexpression.

To further test whether overexpression of *Wt1–KTS* could induce Sertoli to *Foxl2*-expressing granulosa-like cell transformation, *Wt1*^*+/Ctg*^ mice were crossed with *AMH-Cre* transgenic mice. Co-expression of GFP and AMH was observed in *mT-mG;AMH-Cre* testis at E14.5 (Supplementary Fig. S7c, white arrows), indicating that *AMH-Cre* was activated explicitly in Sertoli cells. The mRNA level of *Wt1–KTS* in *Wt1*^*+/Ctg*^*;AMH-Cre* was significantly increased compared with control testes (Supplementary Fig. S7d), confirming the successful overexpression of *Wt1–KTS* in Sertoli cells.

At E16.5, seminiferous tubules were well-organized, and SOX9-positive Sertoli cells (yellow arrows) were located within the seminiferous tubules in both control and *Wt1*^*+/Ctg*^*;AMH-Cre* testes (Supplementary Fig. S8). 3β-HSD-positive Leydig cells (white arrows) were also observed in the interstitium of both control (Supplementary Fig. S8b) and *Wt1*^*+/Ctg*^*;AMH-Cre* (Supplementary Fig. S8e) testes, while no SOX9 (Supplementary Fig. S8g) and 3β-HSD (Supplementary Fig. S8h) signals were noted in control ovaries. FOXL2-positive cells were detected in control ovaries (white arrowheads) but not in control and *Wt1*^*+/Ctg*^*;AMH-Cre* testes (Supplementary Fig. S8). Moreover, the mRNA levels of Sertoli cells-specific genes (e.g., *Sox9*, *Amh*, *Dhh*, and *Fgf9*), steroidogenic cells-specific genes (e.g., *Cyp11a1*, *Star*, *Hsd3b1*, and *Cyp17a1*), and pre-granulosa cells-specific genes (e.g., *Foxl2*, *Rspo1*, *Fst*, and *Wnt4*) were not changed in *Wt1*^*+/Ctg*^*;AMH-Cre* testes (Supplementary Fig. S8j). Furthermore, the cell fate of *Wt1–KTS*-overexpressing Sertoli cells was examined by co-staining of α-SMA and SOX9, α-SMA and FOXL2, and α-SMA and 3β-HSD in *Wt1*^*+/Ctg*^*;AMH-Cre* testes at 2 weeks of age. Seminiferous tubules in *Wt1*^*+/Ctg*^*;AMH-Cre* testes were well-organized, and SOX9-positive Sertoli cells were appropriately located at the periphery region (Supplementary Fig. S9d). Similar to the results observed at the embryonic stage, FOXL2-positive granulosa cells were not detected (Supplementary Fig. S9e), and abundant 3β-HSD-positive steroidogenic cells were present in the interstitium of *Wt1*^*+/Ctg*^*;AMH-Cre* testes (Supplementary Fig. S9f). These results collectively demonstrate that the overexpression of *Wt1–KTS* does not induce transdifferentiation of Sertoli into granulosa-like cells.

In the study, we also observed FOXL2-positive granulosa-like cells in the testes of *Wt1*^*+/Ctg*^*;Sf1-Cre* mouse model. However, we did not observe male-to-female sex reversal, and the differentiation of testes was not affected by *Wt1–KTS* overexpression. The integrity of testicular cords remained intact, and the expression of the SOX9 in Seroli cells was unchanged. Importantly, we did not detect FOXL2-positive cells inside the seminiferous tubules, indicating that the fate of Sertoli cells remained unaltered with *Wt1–KTS* overexpression. Surprisingly, the development of gonads after sex determination, particularly regarding follicle development, was not examined in the study by Gregoire et al. despite the reported male-to-female sex reversal^8^.

The androgens produced by Leydig cells are crucial for the development of the male reproductive system and spermatogenesis. Deficiency in testosterone production has been associated with defects in spermatogenesis in mouse models and human patients^13^. Therefore, the absence of Leydig cells could explain the spermatogenic defects and male infertility observed in our mouse model. In the present study, we observed a blockade in the differentiation of Leydig cells upon overexpression of *Wt1–KTS* in undifferentiated gonadal somatic cells. This finding aligns with our previous research^9–11^, reinforcing the notion that *Wt1* represses the differentiation of Leydig cells. Furthermore, our mouse model also exhibited FOXL2-positive granulosa-like cells, consistent with the recent study by Gregoire et al.^8^. However, the specific origin of these FOXL2-positive cells remained a critical question left unanswered by their research. They concluded that these cells are derived from *Wt1–KTS*-overexpressing Sertoli cells, but they did not conduct lineage tracing experiments to validate this assertion^8^. Additionally, they did not explore the fate of Leydig cells following *Wt1–KTS* overexpression. Given that a subset of Leydig cells shares a common origin with Sertoli cells and that *Wt1* is a critical factor in their differentiation, it becomes imperative to ascertain whether Leydig cells undergo normal differentiation upon *Wt1–KTS* overexpression.

As proposed in the schematic diagram illustrated in Fig. 1g, it is widely acknowledged that both Sertoli and Leydig cells originate from *Wt1*-positive progenitor cells within undifferentiated gonads^14^. During sex determination, a subset of gonadal somatic cells retains *Wt1* expression and initiates the expression of *Sry*, leading to their differentiation into Sertoli cells. Conversely, for reasons yet to be fully understood, a small fraction of somatic cells lose *Wt1* and do not express *Sry*, consequently undergoing differentiation into Leydig cells. In our hypothesis, we postulate that if *Wt1–KTS* is overexpressed in all progenitor cells, the differentiation of Leydig cells would be impeded, while the differentiation of Sertoli cells would remain unaffected. Furthermore, the progenitors of Leydig cells would be unable to differentiate into Sertoli cells under conditions of *Wt1–KTS* overexpression due to the absence of pro-testis factors such as *Sry* and *Sox9*. Instead, they would likely differentiate into granulosa cells because Sertoli and granulosa cells share a common progenitor pool, and the absence of *Sry* or *Sox9* prompts the differentiation of gonadal somatic cells into granulosa cells in male gonads^15^. However, our study did not provide direct evidence to support this conclusion due to the inability to overexpress *Wt1–KTS*, specifically in Leydig cell progenitors. Nonetheless, crucial findings emerged from our research and we observed the transformation of Leydig cells into FOXL2-positive granulosa cells following the overexpression of *Wt1–KTS* specific in Leydig cells after sex determination. By contrast, the specific overexpression of *Wt1–KTS* in Sertoli cells after sex determination did not disrupt testis development, and no FOXL2-positive cells were detected. Based on these data, we inferred that the FOXL2-positive granulosa cells originated from Leydig cell progenitors rather than Sertoli cells. These findings provide valuable insights into the cellular dynamics underlying gonadal development and differentiation.

## Supplementary information

Supplementary information
