# Supplementary material for: The −KTS isoform of Wt1 induces the transformation of Leydig cells into granulosa-like cells
Source: Cell Discov. 2024 Nov 12;10:113. doi: 10.1038/s41421-024-00732-6 (PMC11555372; doi:10.1038/s41421-024-00732-6)
Supplement: Supplementary file 1 — Supplementary information [file 41421_2024_732_MOESM1_ESM.pdf]

Supplementary figure 1

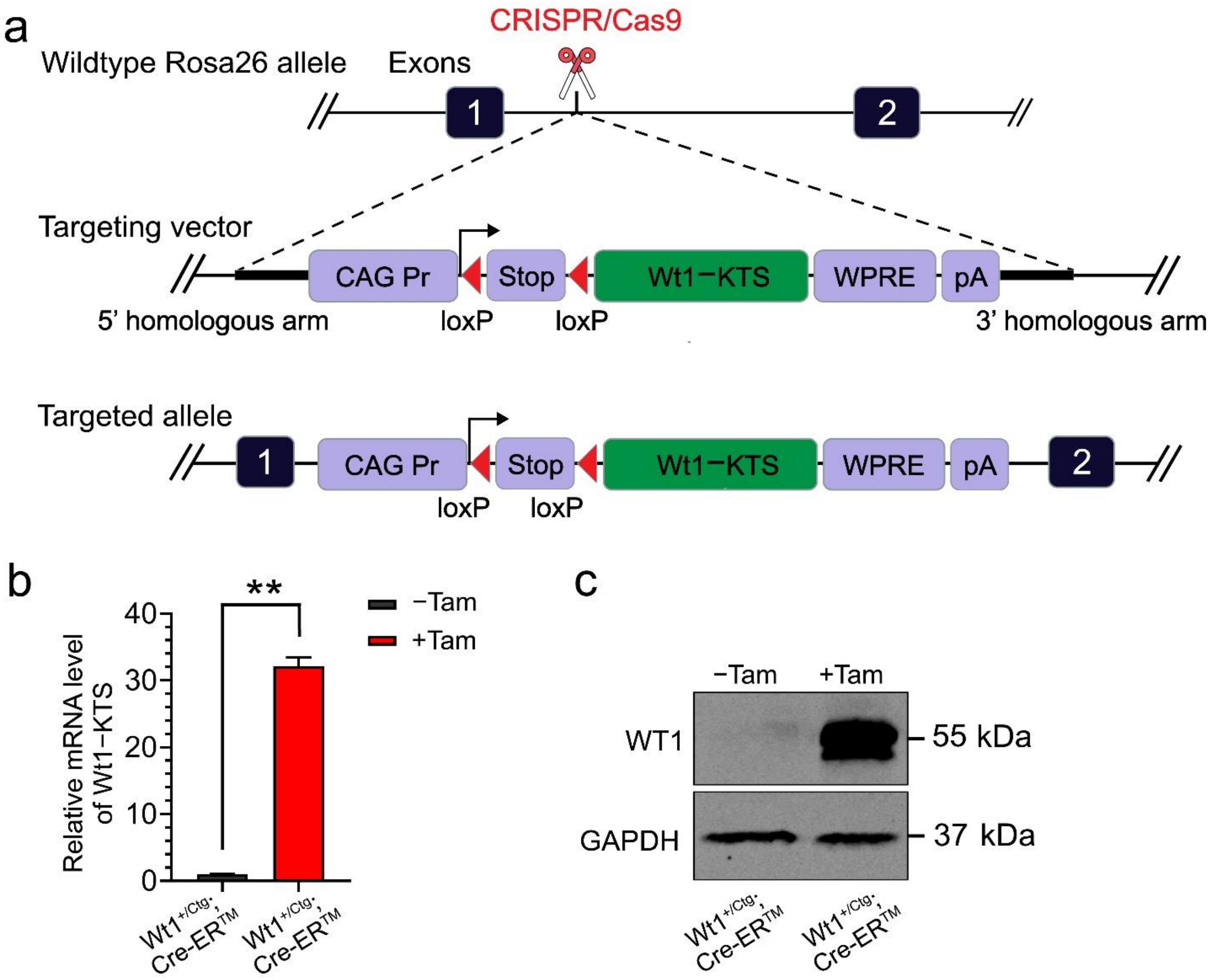

Supplementary figure 2

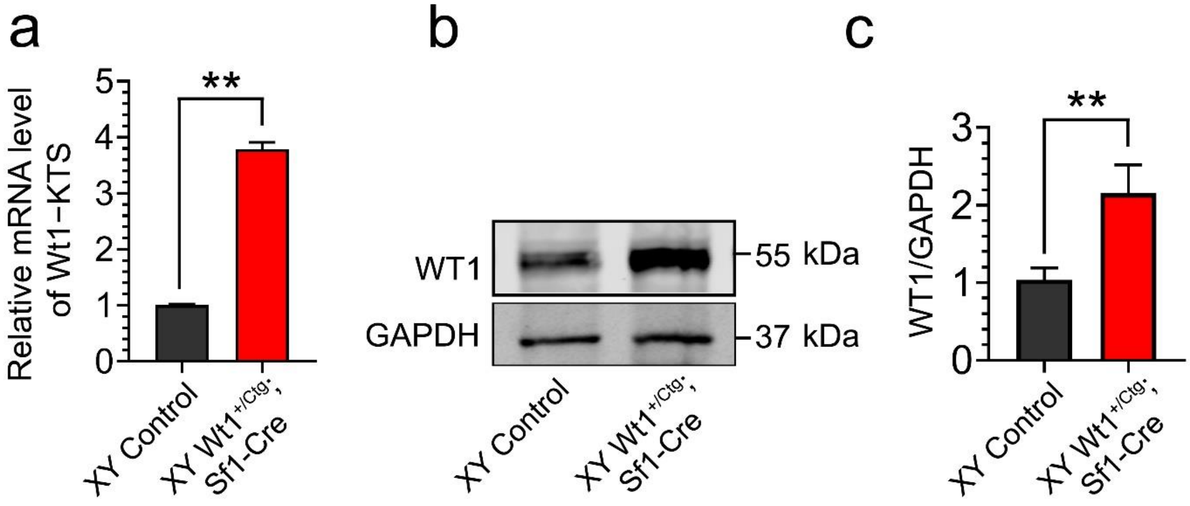

Supplementary figure 3

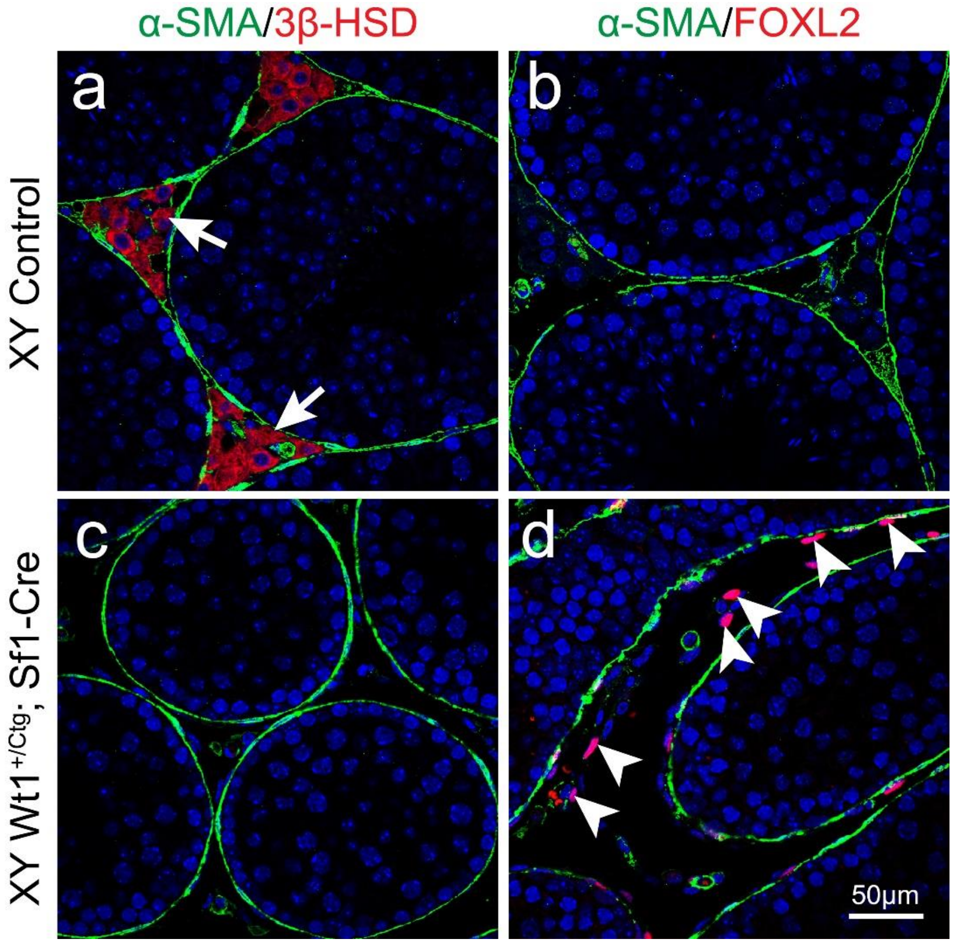

Supplementary figure 4

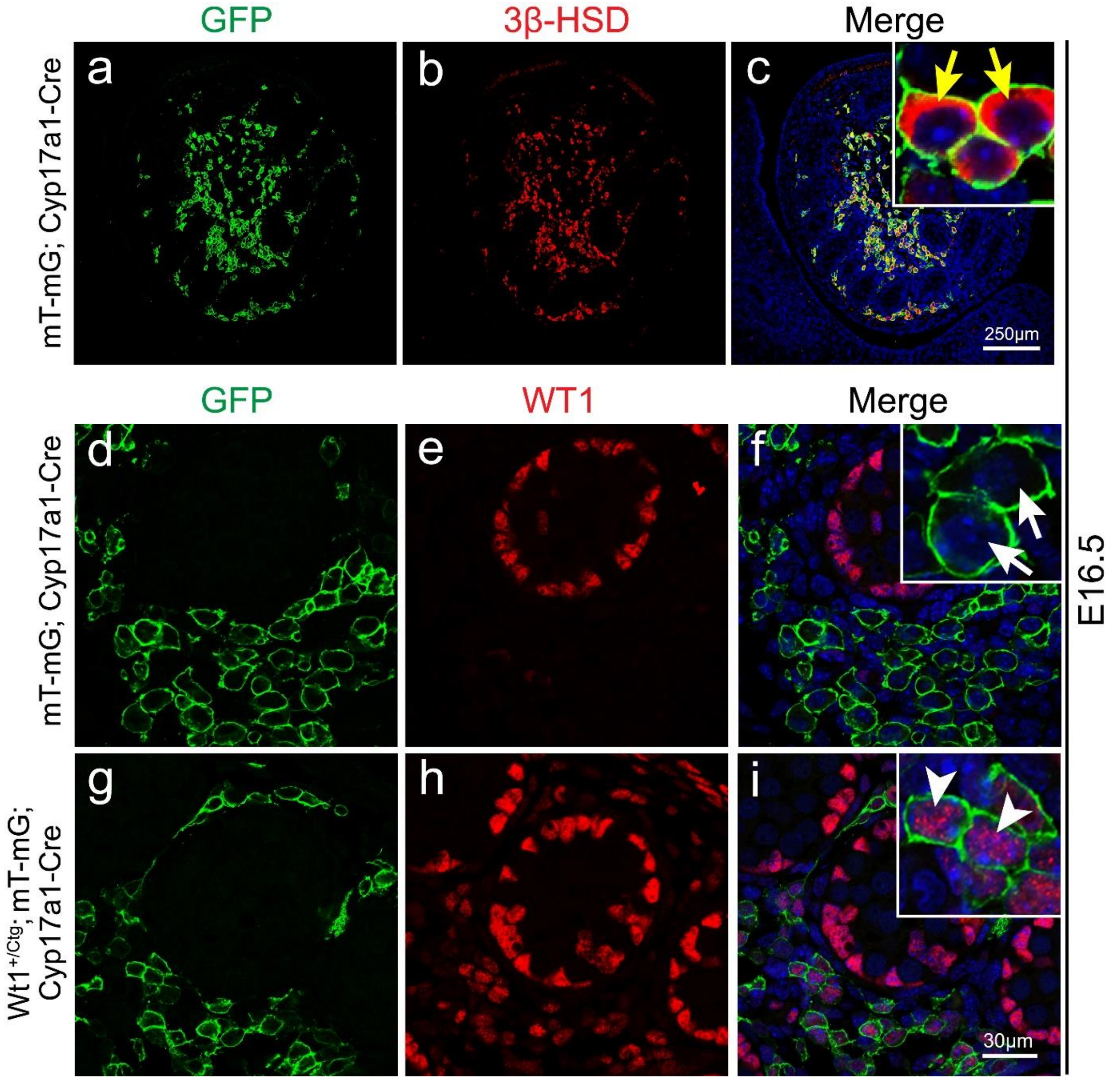

Supplementary figure 5

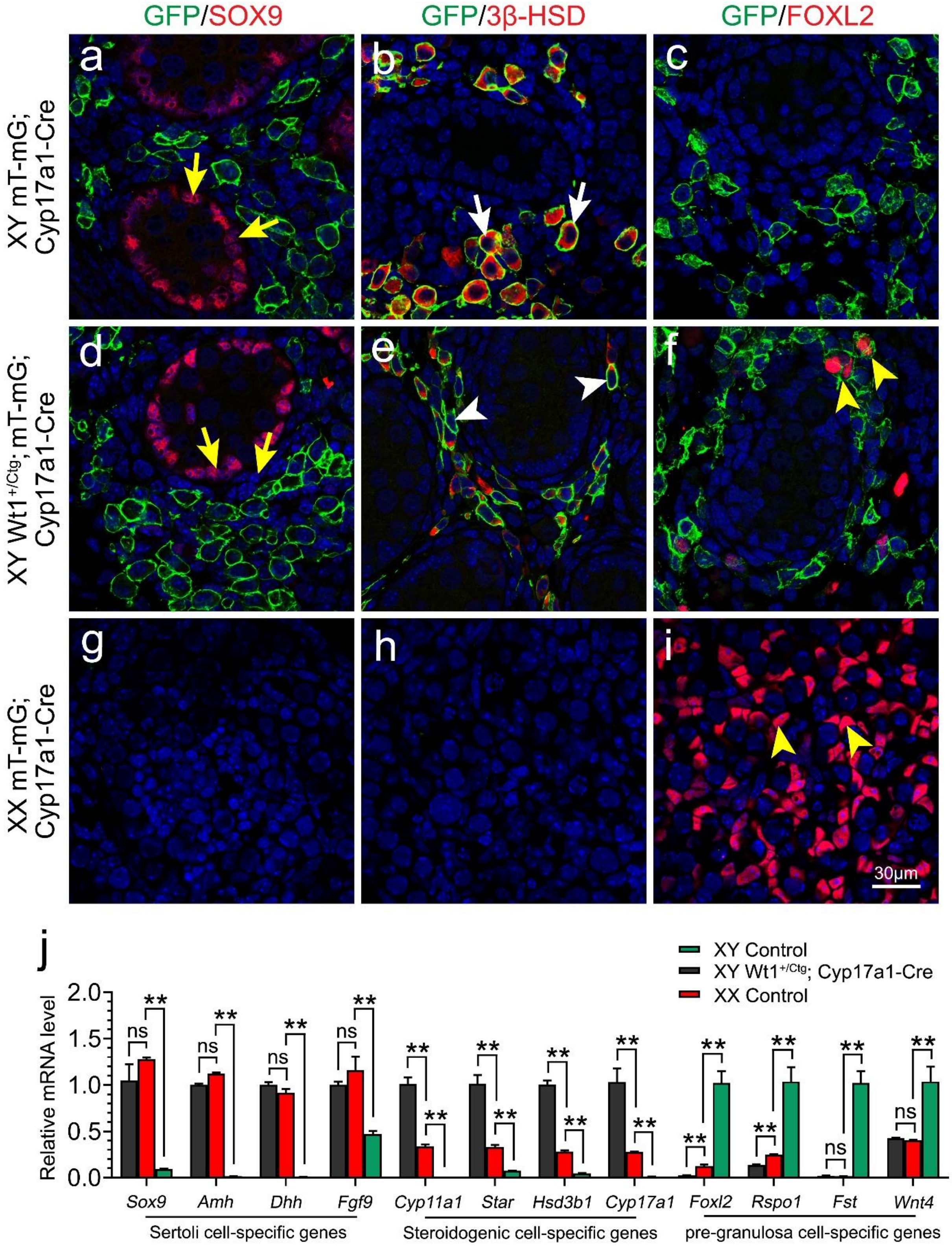

Supplementary figure 6

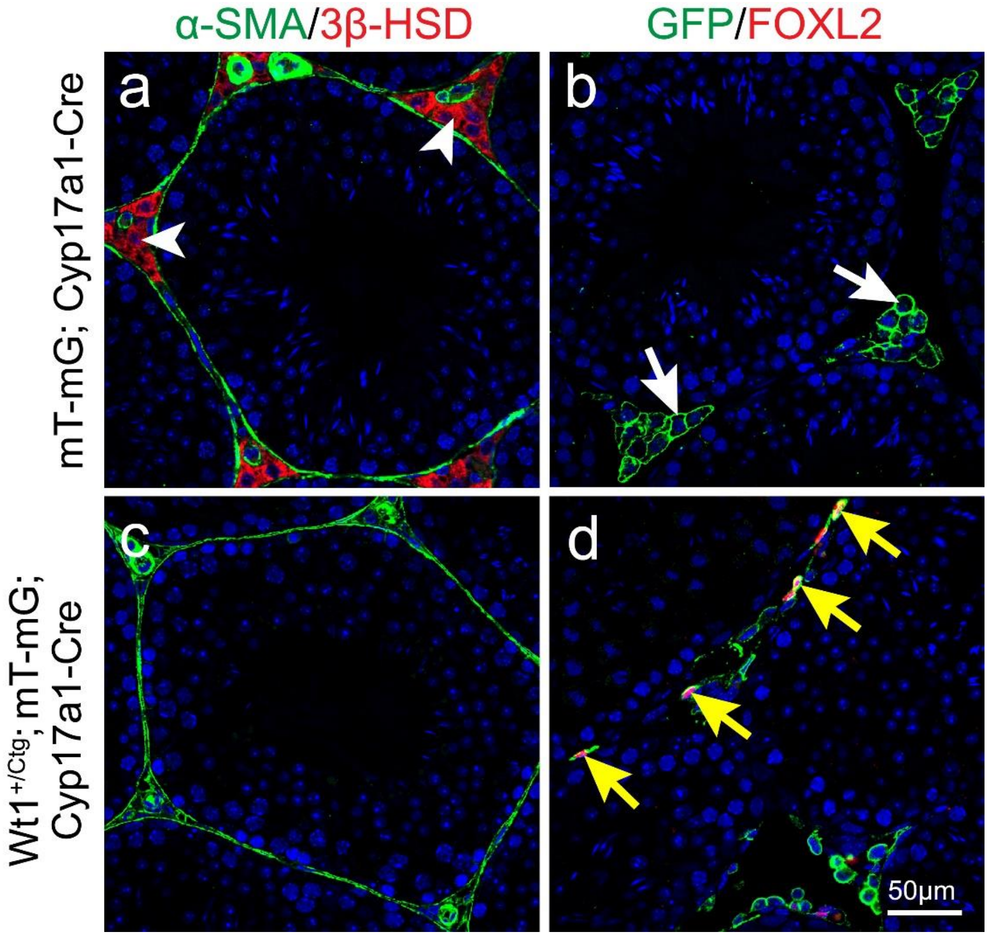

Supplementary figure 7

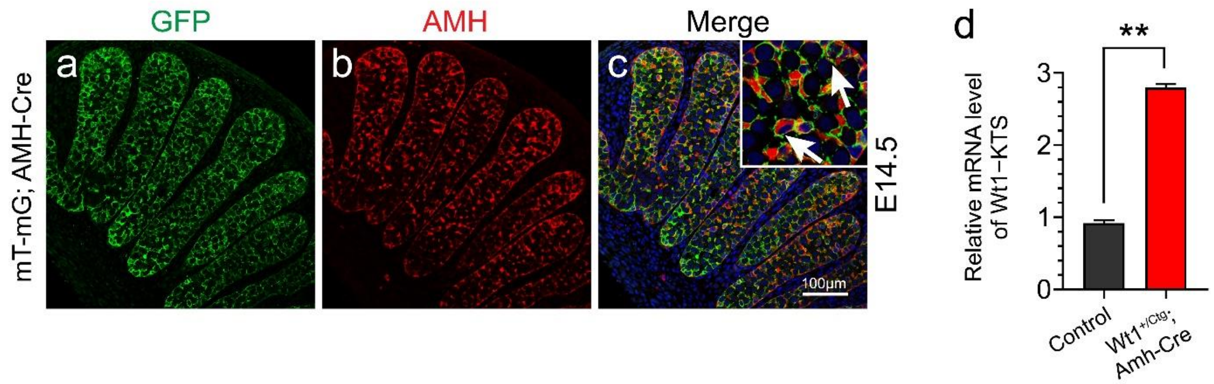

Supplementary figure 8

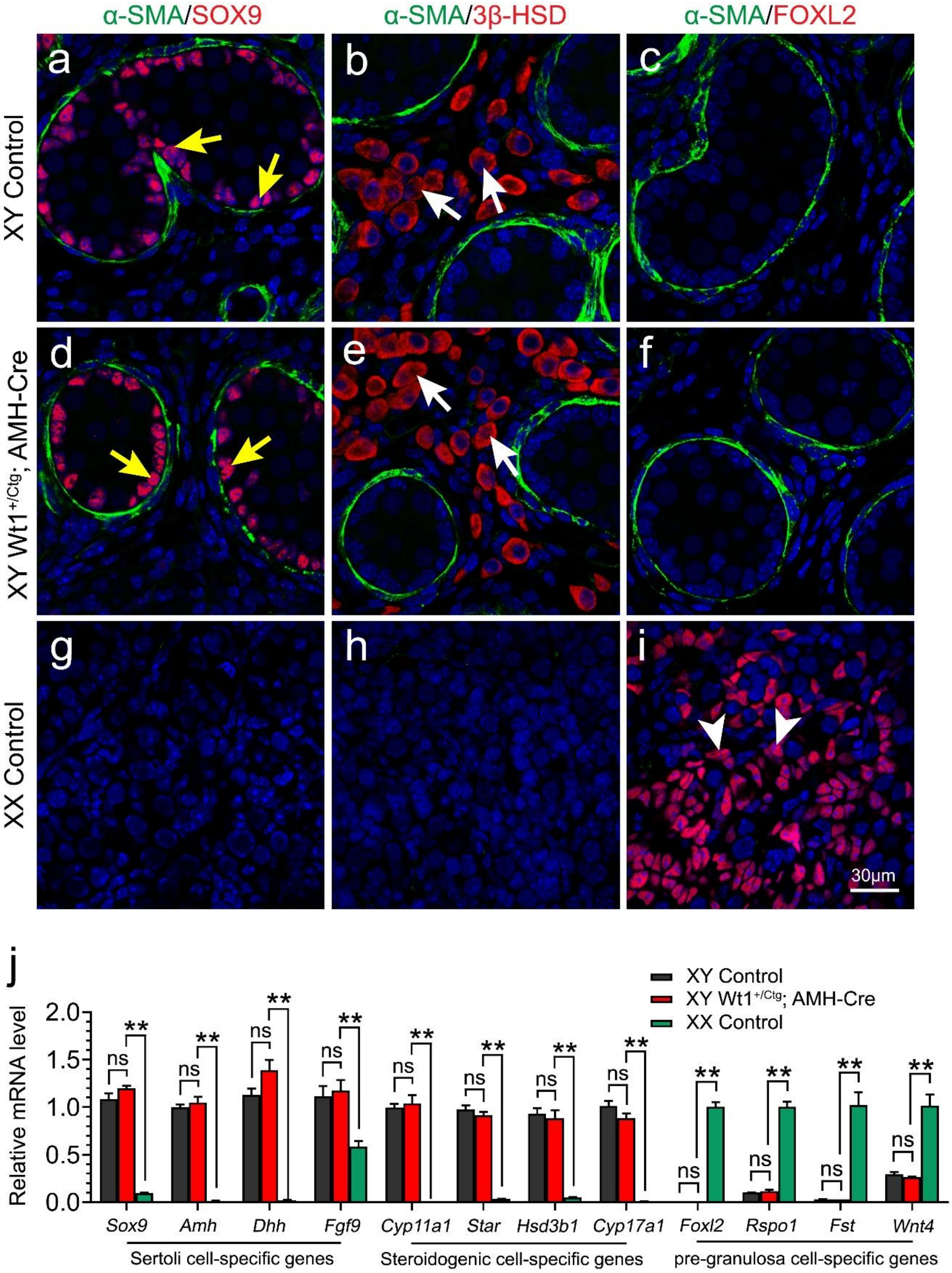

Supplementary figure 9

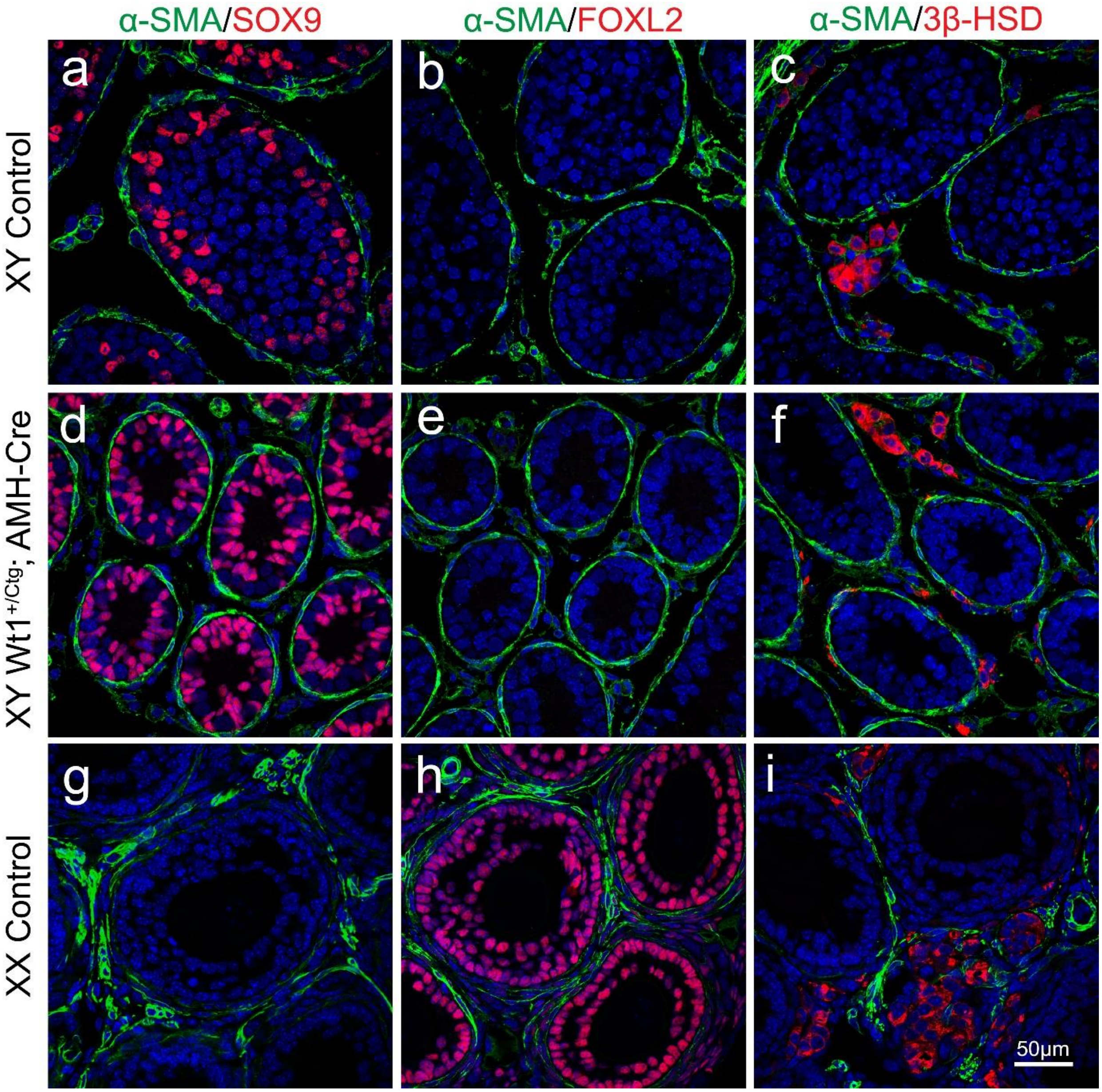

Supplementary table 1

Name	Primer/sgRNA sequnces (5’-3’)
Wt1$^{+/Ctg}$-Forward	CTTCAGCTGTCGGTGGCACAGTT
Wt1$^{+/Ctg}$-Reverse	AGAAGCCAGGAGGCAGCAGAGAA
Wt1$^{+/Ctg}$-sgRNA	CCGCGCGCCCCTGCGCAACG TGG
Cre-ER^{TM}-Forward	CGA TGC AAC GAG TGA TGA GG
Cre-ER^{TM}-Reverse	GCA TTG CTG TCA CTT GGT CGT
Sf1-Cre-Forward	TCCAATTTACTGACCGTACACCAA
Sf1-Cre-Reverse	CCTGTACCTGGCAATTTCGGCTA
Cyp17a1-Cre-Forward	CCGCGCTGGAGTTTCAATAC
Cyp17a1-Cre-Reverse	TGGGTCTATAGAGGGAAAGATCAGG
AMH-Cre-Forward	TCCAATTTACTGACCGTACACCAA
AMH-Cre-Reverse	CCTGTACCTGGCAATTTCGGCTA
mT-mG-Forward1	CTCTGCTGCCTCCTGGCTTCT
mT-mG-Reverse1	CGAGGCGGATCACAAGCAATA
mT-mG-Reverse2	TCAATGGGCGGGGGTCGTT

Supplementary table 2

Gene	Forward primer (5’-3’)	Reverse primer (5’-3’)
Wt1–KTS	CATACCAGTGTGACTTCAAGGACT	AGGGCTTTTCACCTGTATGA
Hsd17b3	ATGGCATCGGGAAAGCCTAT	CTCTTCTGCAATGGTCTGTAGC
Fshr	TGCTCTAACAGGGTCTTCCTC	TCTCAGTTCAATGGCGTTCCG
Fgf9	TGCAGGACTGGATTTCATTTAG	CCAGGCCCACTGCTATACTG
Clu	TGAAGGGCCAGTGTGAAAAGT	TGAACAGTCCACAGACAAGATCTC
Dmrt1	GGAGTCTCCCAGCACCTTACG	TCTGCCACTGGTTTCCAGTCT
Sox9	GCATCTGCACAACGCGG	AGCCTCCAGAGCTTGCCC
Amh	TGGTGCTAACCGTGGACTTC	AGCCAAATAGAAAGGCTTGCA
Dhh	GGACCTCGTACCCAACTACAA	CGATGGCTAGAGCGTTCACC
Erbb4	CAGCGCTTCTCAGTCAGTGT	CTGCTGTTCCAGGTCAGAGA
Shbg	CCAAAATCAGCAAACCCCATT	CTGGATCCCAGGTTCGAAAC
Cyp26b1	ACATCCACCGCAACAAGC	GGGCAGGTAGCTCTCAAGTG
Foxl2	ACAACACCGGAGAAACCAGAC	CGTAGAACGGGAACTTGGCTA
Rspo1	CGACATGAACAAATGCATCA	CTCCTGACACTTGGTGCAGA
Wnt4	AGACGTGCGAGAAACTCAAAG	GGAACTGGTATTGGCACTCCT
Fst	TGGATTAGCCTATGAGGGAAAG	TGGAATCCCATAGGCATTTT

Bmp2	GCTAGATCTGTACCGCAGGCACTCA	TCCCACTCATCTCTGGAAGTTCCTC
Esr2	TGTGCTATGGCCAACTTCTG	AGTAACAGGGCTGGCACAAC
Cyp11a1	CCAGTGTCCCCATGCTCAAC	TGCATGGTCCTTCCAGGTCT
Cyp17a1	GATCTAAGAAGCGCTCAGGCA	GGGCACTGCATCACGATAAA
Hsd3b1	CTCAGTTCTTAGGCTTCAGCAATTAC	CCAAAGGCAAGATATGATTTAGGA
Star	CCGGAGCAGAGTGGTGTCA	CAGTGGATGAAGCACCATGC
Nr5a1	AGGTGTCGGGCTACCACTAC	CCACCCCGCATTCGATCAG
Lhr	AATGAGTCCATCACGCTGAAAC	CCTGCAATTTGGTGGAAGAGA
EH	GGACATCCACTTCATCCACGT	AGCAAGGGCTTTGGAGTGC
Gapdh	GTCATTGAGAGCAATGCCAG	GTGTTGCTACCCCCAATGTG

Supplementary table 3

Antibody name	Reference	Dilution	Source
SOX9	Ab5535	1:200	Millipore
FOXL2	ab246511	1:100	Abcam
WT1	ab89901	1:200	Abcam
CYP17A1	94004S	1:200	Cell signaling technology
3β-HSD	sc30820	1:200	Santa Cruz
AMH	sc6886	1:200	Santa Cruz
GFP	ab13970	1:1000	Abcam
α-SMA	ab5694	1:200	Abcam
GAPDH	AF5009	1:1000	Beyotime

Supplementary Fig 1. Generation of the *Wt1*$^{+/Ctg}$ mouse model. a Schematic diagram of strategy for generating *Wt1−KTS* knock-in mouse model. **b** The mRNA level of *Wt1−KTS* in *Wt1*$^{+/Ctg}$*; Cre-ER*^{TM} MEFs was significantly increased after tamoxifen treatment (*n* = 6). **c** The protein level of WT1 was dramatically increased in *Wt1*$^{+/Ctg}$*; Cre-ER*^{TM} MEFs after tamoxifen treatment. Tam, tamoxifen. Data are presented as mean ± SEM. *P <0.05; **P <0.01.

Supplementary Fig 2. The expression of *Wt1−KTS* was significantly increased in the testis of *Wt1*$^{+/Ctg}$*; Sf1-Cre* mice at E12.5. a The mRNA level of *Wt1−KTS* was significantly increased in testes of *Wt1*$^{+/Ctg}$*; Sf1-Cre* mice at E12.5 compared to control testes (*n* = 6). **b-c** The protein level of WT1 was significantly increased in *Wt1*$^{+/Ctg}$*; Sf1-Cre* testes (*n* = 3). Data are presented as mean ± SEM. *P <0.05; **P <0.01.

Supplementary Fig 3. FOXL2-positive granulosa-like cells were detected in the testis interstitium of adult *Wt1*$^{+/Ctg}$*; Sf1-Cre* mice. The seminiferous tubules were outlined by α-SMA (Green). **a**, **c** 3β-HSD-positive Leydig cells (**a**, white arrows) were observed in control testes but not in testes of *Wt1*$^{+/Ctg}$*; Sf1-Cre* mice (**c**). **b, d** A few FOXL2-positive cells were noted in the interstitium of *Wt1*$^{+/Ctg}$*; Sf1-Cre* (**d**, white arrowheads) but not in control testes (**b**).

Supplementary Fig 4. WT1−KTS protein was detected in Leydig cells of *Wt1*$^{+/Ctg}$*; Cyp17a1-Cre* mice at E16.5. *Wt1−KTS* was overexpressed in Leydig cells by crossing *Wt1*$^{+/Ctg}$*; mT-mG* mice with *Cyp17a1-Cre* mice. **c**, **f** The expression of GFP, WT1, and 3β-HSD in testes of *mT-mG; Cyp17a1-Cre* and *Wt1*$^{+/Ctg}$*; mT-mG; Cyp17a1-Cre* mice was examined by immunofluorescence. GFP signal was detected in testes of *mT-mG; Cyp17a1-Cre* mice (**c**, yellow arrows) and

colocalized with 3β-HSD, but no WT1 signal was detected in GFP-positive cells (**f**, white arrows). **i** The expression of WT1 was expressed in not only Sertoli cells but also GFP-positive Leydig cells in testes of *Wt1*$^{+/Ctg}$*; mT-mG; Cyp17a1-Cre* mice (white arrowheads).

Supplementary Fig 5. Overexpression of *Wt1−KTS* induced Leydig cells to granulosa-like cells transformation. *Wt1−KTS* was overexpressed in Leydig cells by crossing *Wt1*$^{+/Ctg}$*; mT-mG* mice with *Cyp17a1-Cre* mice. The expression of SOX9, GFP, 3β-HSD, and FOXL2 in testes and ovaries of *mT-mG; Cyp17a1-Cre* mice, and testes of *Wt1*$^{+/Ctg}$*; mT-mG; Cyp17a1-Cre* mice at E16.5 was examined by immunofluorescence. **a, d, g** SOX9-positive cells (red, yellow arrows) were detected in the testicular cords of *mT-mG; Cyp17a1-Cre* (**a**) and *Wt1*$^{+/Ctg}$*; mT-mG; Cyp17a1-Cre* (**d**) mice but not in ovaries of *mT-mG; Cyp17a1-Cre* mice (**g**). **a, d, g** GFP signal was detected in the testicular interstitium of *mT-mG; Cyp17a1-Cre* (**a**) and *Wt1*$^{+/Ctg}$*; mT-mG; Cyp17a1-Cre* (**d**) mice but not in ovaries of *mT-mG; Cyp17a1-Cre* mice (**g**). 3β-HSD (red) was expressed in testes of *mT-mG; Cyp17a1-Cre* mice and colocalized with GFP (B, white arrows). **e** 3β-HSD was also detected in testes of *Wt1*$^{+/Ctg}$*; mT-mG; Cyp17a1-Cre* mice and colocalized with GFP (white arrowheads), whereas the expressive level was significantly reduced. **c, i** large number of FOXL2-positive cells (red) were noted in control ovaries (**i**, yellow arrowheads) but not in control testes (**c**). **f** FOXL2 signal (yellow arrowheads) was detected in testes of *Wt1*$^{+/Ctg}$*; mT-mG; Cyp17a1-Cre* mice and colocalized with GFP. **j** The mRNA level of Sertoli cell-specific genes, such as *Sox9*, *Amh*, *Dhh*, and *Fgf9*, was not significantly changed in testes of *Wt1*$^{+/Ctg}$*; mT-mG; Cyp17a1-Cre* mice, but steroidogenic cell-specific genes, such as *Cyp11a1*, *Star*, *Hsd3b1*, and *Cyp17a1* were significantly decreased compared with control testes ($n = 6$). The mRNA level of *Foxl2* and *Rspo1*

was up-regulated in *Wt1*$^{+/Ctg}$*; Cyp17a1-Cre* testes, whereas *Fst* and *Wnt4* were not increased in *Wt1*$^{+/Ctg}$*; Cyp17a1-Cre* teste (n = 6). Data are presented as mean ± SEM. **P* <0.05; ***P* <0.01.

Supplementary Fig 6. FOXL2-positive cells were observed in the testes of adult *Wt1*$^{+/Ctg}$*; Cyp17a1-Cre* mice. a, c The seminiferous tubules were outlined by α-SMA (Green). 3β-HSD-positive Leydig cells were observed in the interstitium of control testes (**a**) but not in *Wt1*$^{+/Ctg}$*; mT-mG; Cyp17a1-Cre* testis (**c**). **b, d** No GFP and FOXL2 double-positive cells were noted in control testes (**b**, white arrows), but several GFP and FOXL2 double-positive cells were observed in *Wt1*$^{+/Ctg}$*; mT-mG; Cyp17a1-Cre* testes (**d**, yellow arrows).

Supplementary Fig 7. Overexpression of *Wt1−KTS* in Sertoli cells using *AMH-Cre* transgenic mice. a-c Co-expression of GFP (**a**, green) and Sertoli cell marker AMH (**b**, red) in E14.5 *mT-mG; Sf1-Cre* gonads (**c**, white arrows). **d** The mRNA level of *Wt1−KTS* was significantly up-regulated in *Wt1*$^{+/Ctg}$*; AMH-Cre* compared to that level measured in control testes (n = 6). Data are presented as mean ± SEM. **P* <0.05; ***P* <0.01.

Supplementary Fig 8. No FOXL2-positive cell was detected in the testis of *Wt1*$^{+/Ctg}$*; AMH-Cre* mice at E16.5. *Wt1−KTS* was overexpressed in Sertoli cells by crossing *Wt1*$^{+/Ctg}$*; mT-mG* mice with *AMH-Cre* mice. **a, d, g** The expression of SOX9, 3β-HSD, α-SMA, and FOXL2 in control testes, control ovaries, and testes of *Wt1*$^{+/Ctg}$*; AMH-Cre* mice at E16.5 were examined by immunofluorescence. SOX9-positive Sertoli cells (red, yellow arrows) were observed in testicular cords of control (**a**) and *Wt1*$^{+/Ctg}$*; AMH-Cre* mice (**d**) but not in control ovaries (**g**). **b, e, h** 3β-HSD-positive Leydig cells (red, white arrows) were observed in the testes of control (**b**) and

Wt1$^{+/Ctg}$*; AMH-Cre* mice (**e**) but not in control ovaries (**h**). **c, f, i** FOXL2-positive granulosa cells (red, white arrowheads) were observed in control ovaries (**i**) but not in control testes (**c**) and *Wt1*$^{+/Ctg}$*; AMH-Cre* testes (**f**). **j** The mRNA levels of Sertoli cell-specific genes, steroidogenic cell-specific genes, and granulosa cell-specific genes were examined by RT-PCR (n = 6). Data are presented as mean ± SEM. *P <0.05; **P <0.01.

Supplementary Fig 9. No feminized characteristics were detected in *Wt1*$^{+/Ctg}$*; AMH-Cre* mice at 2 weeks of age. The seminiferous tubules and follicles were outlined by α-SMA (Green). **a, d, g** SOX9-positive Sertoli cells were normally located in seminiferous tubules in both control (**a**) and *Wt1*$^{+/Ctg}$*; AMH-Cre* (**d**) testes and no signal was observed in control ovaries (**g**). **b, e, h** Numerous FOXL2-positive granulosa cells were detected in control ovaries (**h**) but not in control (**b**) and *Wt1*$^{+/Ctg}$*; AMH-Cre* (**e**) testes. **c, f, i** 3β-HSD protein was detected in the interstitium in control testes (**c**), *Wt1*$^{+/Ctg}$*; AMH-Cre* (**f**) testes, and control ovaries (**i**).

Supplementary table 1. List of primers of PCR and sgRNA sequences.

Supplementary table 2. List of primers used for real-time PCR to assess the relative mRNA level of target genes.

Supplementary table 3. List of antibodies.

Supplementary materials and methods

Mice

All animal experiments were conducted following the protocols approved by the Institutional Animal Care and Use Committee of the Institute of Zoology, Chinese Academy of Sciences (CAS; SYXK 2018-0021). All mice were derived from a C57/ICR mixed background and housed under specific pathogen-free (SPF) laboratory conditions. *Wt1*$^{+/Ctg}$ mice were mated with mice carrying the *Cre-ER*^{TM} (*14*), *Sf1-Cre*[1,2], *Cyp17a1-Cre*, *AMH-Cre*[3], and *mT-mG* transgenic[4] mice to generate *Wt1*$^{+/Ctg}$*; Cre-ER*^{TM}, *Wt1*$^{+/Ctg}$*; Sf1-Cre*, *Wt1*$^{+/Ctg}$*; mT-mG; Cyp17a1-Cre*, and *Wt1*$^{+/Ctg}$*; mT-mG; AMH-Cre* mice. Detailed information on the generation of the *Wt1*$^{+/Ctg}$ knock-in (KI) mouse model was provided in Supplementary Fig. S1. The mice were genotyped using DNA isolated from tail biopsies through polymerase chain reaction (PCR) and Sanger sequencing. The specific primers used for PCR are listed in Supplemental table S1.

Histological analysis

Tissues were initially fixed in 4% paraformaldehyde (PFA) for 18–24 hours (h) and transferred to 70% ethanol. Following fixation, the samples were embedded in paraffin, and 5-µm sections were cut and then mounted on glass slides. After deparaffinization and rehydration, the sections were processed with hematoxylin and eosin (H&E) staining. Images were captured with a Nikon Digital sight (DS)-Ri1 charge-coupled device (CCD) camera.

Immunohistochemistry (IHC) analysis

IHC was performed as described previously[5]. Briefly, tissues were fixed in 4% paraformaldehyde (PFA) for 18–24 hours (h) and then transferred to 70% ethanol. Fixed samples were embedded in

paraffin, and 5-μm sections were cut and then mounted on glass slides. After deparaffinization and rehydration, the sections were processed with IHC staining. IHC staining was conducted using a Vectastain ABC (avidin-biotin-peroxidase) kit (Vector Laboratories, Burlin-Game, CA) as recommended. The stained sections were examined using Nikon Microscopy, and images were captured with a Nikon DS-Ri1 CCD camera.

Immunofluorescence (IF) analysis

Gonads samples were fixed, embedded, sectioned, deparaffinized, and rehydrated as described for IHC. After antigen retrieval, the sections were incubated with 0.3% Triton X-100 for 10 minutes for antigen retrieval and incubated with blocking buffer (BSA, 5% w/v) for 1 hr at room temperature (RT). Subsequently, the sections were incubated with the diluted primary antibodies. After washing three times with phosphate-buffered saline (PBS), the sections were incubated with corresponding Fluorescein isothiocyanate (FITC), CyTM3, and Tetrametrylrhodarnine Isothiocyanate (TRITC)-conjugated second antibodies (Jackson ImmunoResearch) and 4, 6-diamidino-2-phenylindole (DAPI) for 1 hr at RT (room temperature). Finally, images were captured with a Zeiss LSM 880 confocal microscope (Carl Zeiss Inc, Thornwood, NY). The fluorescence intensity of the DNA methylation signal was quantified by using Fiji ImageJ software.

Real-time PCR (RT-PCR) analysis

Total RNA was isolated from gonads using a RNeasy Kit (Aidlab, RN28) according to the manufacturer-recommended protocol. The total RNA concentration was measured using a NanoDrop 2000 (Thermo Fisher Scientific). Subsequently, 2 μg of total RNA was used to synthesize the first-strand cDNA (Abm, G592). Diluted cDNAs were analyzed by RT-PCR using

SYBR Green I Mastermix (Abm, MasterMix-S) with a LightCycler 480 system (Roche). Amplification was performed: denaturation steps at 95 ℃ for 10 minutes, 40 cycles of 95 ℃ for 15 seconds, and 60 ℃ for 1 minute. All gene expression was normalized to *Gapdh*. The relative target gene expression levels were calculated using the formula $2^{-\Delta\Delta CT}$ and specific primers (Supplementary table S2).

Western blot analysis

For mouse embryonic fibroblast (MEF) and testes, total protein was harvested with RIPA Lysis Buffer (Applygen, P1053) supplemented with Complete Mini Protease Inhibitor Cocktail Tablets (Roche Applied Science, 04693116001) and 1 mM Phenylmethanesulfonyl fluoride (PMSF). The homogenates or cell lysates were centrifuged at 13,000 × g for 15 minutes at 4 °C to remove debris, and the protein concentration in the supernatant was determined using the Bio-Rad protein assay kit (Bio-Rad). Equal amounts of protein lysates (approximately 25 μg) were separated by sodium dodecyl sulfate-polyacrylamide gel electrophoresis (SDS-PAGE) and transferred onto nitrocellulose membranes. The membranes were blocked with 5% skim milk following transfer to prevent non-specific binding. The membranes were then incubated overnight at 4 ℃ with the following primary antibody. After thorough washing, the membranes were incubated with secondary antibodies for 1 hour at room temperature. The immunoreactive bands were visualized and detected using the ODYSSEY Sa Infrared Imaging System (LI-COR Biosciences, Lincoln, NE). GAPDH was used as a reference control and that quantitation was performed by normalizing WT1 protein expression levels to those of GAPDH. The antibodies used are listed in Supplemental table S3.

Statistical analysis

All experiments were repeated three times, including at least three independent biological samples. Quantitative data are presented as mean ± SEM. A p-value < 0.05 was considered statistically significant using an unpaired two-tailed Student’ s t-test. Analysis was performed using Prism version 8.0.1 (GraphPad Software).

References

1. Bingham, N. C. et al. *Genesis* **44**, 419-424 (2006).
2. Huang, C. C., Yao, H. H. *BMC Dev Biol* **10**, 66 (2010).
3. Lécureuil, C. et al. *Genesis* **33**, 114-118 (2002).
4. Muzumdar, M. D. et al. *Genesis* **45**, 593-605 (2007).
5. Gao, F. et al. *Proc Natl Acad Sci U S A* **103**, 11987-11992 (2006).
